# Efficacy of Operculectomy in the Treatment of 145 Cases with Unerupted Second Molars: A Retrospective Case–Control Study

**DOI:** 10.3390/dj8030065

**Published:** 2020-07-01

**Authors:** Andrea Abate, Davide Cavagnetto, Andrea Fama, Marco Matarese, Francesca Bellincioni, Fausto Assandri

**Affiliations:** 1 Department of Biomedical, Surgical and Dental Sciences, School of Dentistry, University of Milan, 20100 Milan, Italy; andrea.abate@unimi.it (A.A.); davide.cavagnetto@unimi.it (D.C.); andrea.fama@unimi.it (A.F.); francesca.bellincioni@unimi.it (F.B.); 2 Fondazione IRCCS Cà Granda, Ospedale Maggiore Policlinico, 20100 Milan, Italy; 3 Department of Biomedical, Odontostomatological Sciences and Morphological and Functional Images, School of Dentistry, University of Messina, Via Consolare Valeria 1, 98125 Messina, Italy; marco.matarese@unimi.it

**Keywords:** eruption, impacted second molars, surgical exposure, retention

## Abstract

The aim of this study is to assess whether operculectomy in patients with retained second molars eases spontaneous tooth eruption in respect to untreated controls. Two hundred and twenty-two patients with delayed eruption of at least one second molar were selected from the archives of the Department of Orthodontics, Milan, Italy. Eighty-eight patients, 40 males and 48 females (mean age 14.8 ± 1.3 years), met the inclusion criteria. Records were then divided into case and control groups. The case group consisted of patients that underwent removal of the overlaying mucosa over second molars (i.e., operculectomy) and the control group consisted of subjects who retained their operculum over an unerupted second molar and were followed for one year without performing any treatment. A total of 145 impacted second molars were considered (75 cases, 70 controls). A risk ratio with 95% confidence interval was used to compare the prevalence of eruption in the two groups. Spontaneous eruption occurred in 93.3% of cases in the operculectomy group (70/75), while in the control group, 10% teeth erupted spontaneously (7/70). Spontaneous eruption in the upper arch occurred in 95.2% of cases among treated patients (40 out of 42), while in the lower arch, spontaneous eruption occurred in 90.9% of cases (30 out of 33). Spontaneous eruption of the upper second molars in the control group occurred in 8.5% of cases (3 out of 35), while in the lower arch, it occurred in 8.5% (3 out of 35). Operculectomy can ease the spontaneous eruption of retained second molars and reduce the chances of inclusion.

## 1. Introduction

Failure of eruption of permanent molars is a relatively rare condition [1]. The etiology is allegedly related to interference with physiological tooth development. Retention of second permanent molars occurs in between 0.03% and 0.58% of mandibular molars and between 0.04% and 0.08% of maxillary molars [1].

Andreasen and Kurol classified failure of eruption under three clinical conditions: impaction that is the stop of the eruption process due to a physical obstruction radiographically or clinically detectable; primary retention that is a disturbance in the eruption process before the tooth has surfaced in the oral cavity; and secondary retention that is a stop in the eruption process after it has already begun, and the tooth had already penetrated the gingiva in absence of any physical obstruction [2,3,4,5].

The etiologies of eruption disorders of maxillary and mandibular molars are allegedly connected to several conditions: mechanical obstructions (hyperdontia, tumors, cysts, mesial eruption and subsequent impaction into the distal aspect of the adjacent tooth); dentoalveolar discrepancy; augmented space between developing second molar and first molars [6]; abnormal eruption path [5]; obstruction of the eruption pathway of the second molar from third molar gem position [6]; a viral infection that somehow affects local innervation and disturbs the physiological eruption process [7,8]; and genetic predisposition [1,9,10,11].

Second permanent molars usually erupt around twelve years of age [12]. The most favorable age to treat eruption disturbances of second molars is between 10 and 14 years of age because the roots are still under development and the third molars are usually gems [13].

Possible treatments include: periodic non-interventional check-ups, surgical uncovering (operculectomy) and extraction [14]. Periodic check-ups are useful to distinguish temporary delays in eruption from permanent failure. Tooth impaction can lead to several complications like the development of cysts and radicular resorption of the roots of adjacent teeth [15,16,17].

Operculectomy, that is the surgical removal of a flap of gum tissue over the partially erupted tooth, is useful in the absence of an alteration of tooth position and angulation and in the presence of a radicular system still under development (indication of a residual eruptive potential) [18,19,20]. Most tooth impactions spontaneously solve themselves when hard and/or soft tissue obstacles are removed.

Operculectomy associated with an orthodontic-assisted eruption is required if the position and angulation of the unerupted tooth would allegedly not allow spontaneous eruption. Third molars are usually extracted in both cases to ease tooth eruption [21,22,23,24]. Second molar auto-transplantation is also a procedure described in the literature [25,26,27].

Extraction of retained second molars is an option when certain conditions coexist: infections, destructive caries, cystic lesions, radicular resorption and periodontal damages to adjacent teeth [28]. Ankylosis of the retained tooth makes extraction the treatment of choice [23,29,30]. Moreover, extraction is indicated if surgical uncovering prognosis is unfavorable such as in cases of excessive root inclination [15,31,32,33].

The age of the patient, dental and periodontal status of the impacted tooth and of the adjacent teeth, occlusal relationship and arch length must be taken into consideration.

The purpose of the study is to analyze two groups of patients with impacted second molars and to evaluate the efficacy of operculectomy in the treatment of patients with primary retention of one or more second molars by comparing unerupted second molars treated with surgical uncovering (operculectomy) with an untreated control group composed of patients whose parents did not give consent to perform operculectomy. Both groups were followed for 12 months.

## 2. Materials and Methods

A retrospective case–control study was performed analyzing records of patients with primary retention of one or more second molars. The study design obtained ethical approval from the competent independent ethics committee on 15 March 2016 (Fondazione IRCCS Ca’Granda, Ospedale Maggiore, Milan, Italy: protocol n.573/15). After obtaining informed consent by patients’ parents, anonymized medical records of patients treated between March 1997 and February 2016 at the Department of Orthodontics of the University of Milan (Fondazione IRCCS Ca’ Granda, Ospedale Maggiore Policlinico Milan) were analyzed for research purposes.

The sample of this study was composed of 222 subjects with a diagnosis of delay of eruption of at least one second molar. Inclusion criteria both in the case and in the control group were: availability of full medical records (clinical examination and radiologic exams) and of photographs from the beginning of treatment to the end of the observation period (12 months); no record of any previous orthodontic treatment; and no coexisting general or dental issue apart from one or more impacted second molar with at least two-thirds of the radicular system already present. Exclusion criteria in both groups were: patients with congenital and dental abnormalities; absence of dental records; previous orthodontic treatment; obstruction of the eruptive path by the third molar position; and root anomalies.

Eighty-eight patients, 40 males and 48 females (mean age 14.8 ± 1.3 years), met the inclusion criteria and were included in the final sample. The total number of impacted/retained second molars was 145. Seventy-five molars were treated with operculectomy and included in the case group. The control group consisted of 70 unerupted second molars that were periodically monitored without performing any treatment. Characteristics of the non-erupted second molar were matched between the case and control group in order to evaluate two homogenous samples. In both groups, second molars were mesially inclined or vertically positioned in most of the cases, and enlarged follicle and bigger crown were less frequently observed. All patients were re-evaluated after 12 months (mean age 15.9 ± 1.7).

### 2.1. Study Procedure

Each retained tooth underwent radiographic evaluation. Dental maturity was evaluated according to Demirijan et al. [34] The assessment of tooth inclination was performed on the panoramic radiographies according to Evans [35]. Teeth were considered mesio-tilted when the angle was more than 40 degrees, vertically positioned if the angle ranged between 40 and 20 degrees and distally inclined if the angle was less than 20 degrees [5,8].

**Figure 1:**
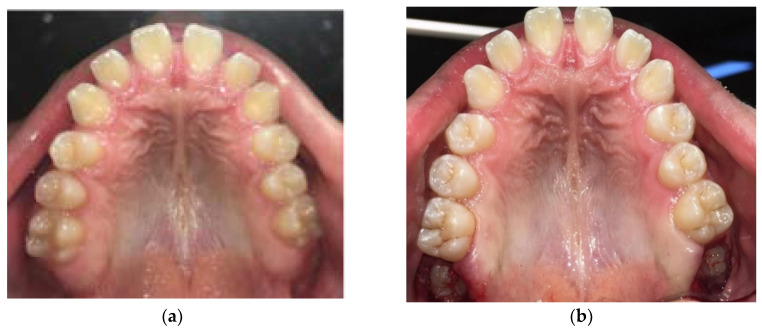

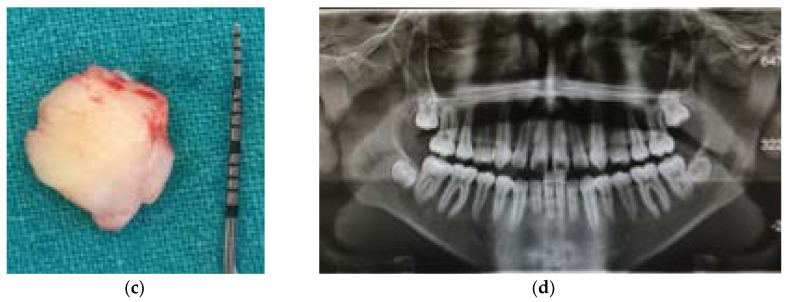
Example of a case treated with operculectomy. (**a**) Pre-surgical occlusal vision; (**b**) post-surgical occlusal vision; (**c**) example of the amount of soft tissue being removed in each side; (**d**) example of pre-treatment orthopantomography.

In the first group, patients underwent surgical removal of the mucosa (operculectomy) covering retained teeth. The area was treated with 2% lignocaine hydrochloride solution with adrenaline (1:80,000). Patients received no antibiotics prior to the surgery. Local antimicrobial prophylaxis was performed by rinsing with 0.2% chlorhexidine gluconate before surgical excision. The surgical excision of the flap of gum overlying second molars (Figure 1a–c) was performed with a surgical scalpel blade No 10. The control group consisted of patients whose parents refused to perform operculectomy. They were not treated but only followed for 12 months. Outcomes after treatment and outcomes without treatment were evaluated. Treatment outcome was considered successful if spontaneous eruption occurred within 1 year after surgical uncovering. Occlusion was considered successful if the tooth erupted vertically and the occlusal surface of the retained tooth was 2 mm or less from the occlusal plane.

### 2.2. Statistical Analysis

Statistical analysis was performed using Microsoft Excel^®^ 2013 (Microsoft Corporation, Redmond, WA, USA) and SPSS v. 21.0 (IBM, Chicago, IL, USA).

The prevalence of eruption of the second molars was compared between the two groups by calculating the risk ratio (RR) with a confidence interval of 95% (95% CI). The chi-squared test was used for testing relationships in cases of an association between 2 dichotomous variables. A statistically significance difference was set at *p* value < 0.05.

## 3. Results

No complications occurred apart from mild tenderness in the area where operculectomy was performed in the first week after the intervention. Eruption occurred in 93.3% of teeth in the treatment group (70/75) after surgical removal of the mucosa, while in the control group, only 10% of teeth erupted (7/70). In the case group, following operculectomy, the eruption in the upper arches occurred in 95.2% of cases (40 out of 42), while in the lower arches, the eruption occurred in 90.9% of cases (30 out of 33). In the control group, the spontaneous eruption of the upper second molars occurred only in 8.5% of cases (3 sites out of 35), while lower molars that spontaneously erupted occurred only in 8.5% of cases (3 sites out of 35).

Percentages of eruption of the second permanent molars, in the case group and in the control group, divided between the upper and lower arches, are shown in Table 1.

The chi-squared test showed a statistically significant difference between the treatment group and the control group (Table 1). In the treatment group, as reported in Table 1, teeth were 10 times more likely to spontaneously erupt than in the control group. The number of permanent second molars that erupted after surgery, in the upper and lower arch, observed in the treatment group was 40/42 and 30/33, respectively. By contrast, in the control group, only 3 molars out of 35 erupted in the oral cavity both in the upper and in the lower arch.

**Figure 2:**
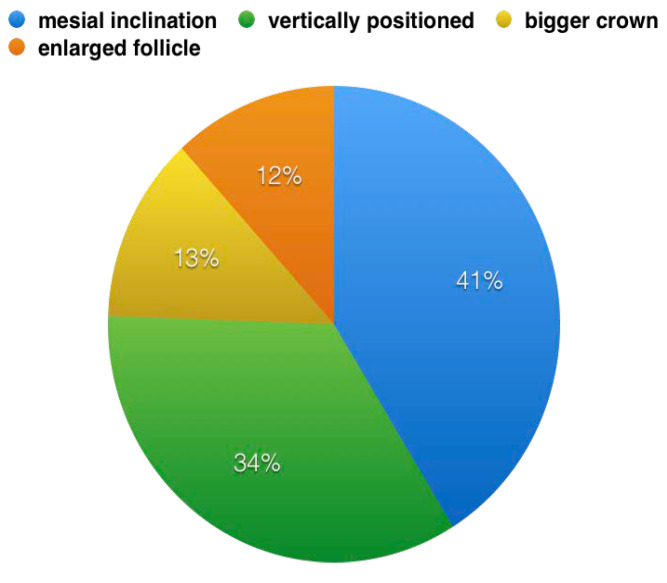
Characteristics of the non-erupted molars.

Among unerupted teeth, 23 were positioned with a mesial inclination (41%), 13 were positioned vertically (34%), 8 showed a bigger crown compared with the contralateral (13%) and 10 showed an enlarged follicle compared with a normal crown follicle (12%) (Figure 2).

## 4. Discussion

Even if delayed eruption of second molars in the upper and lower arch is a relatively rare issue, it causes several problems. Even when the second molar spontaneously erupts, it delays the beginning of the orthodontic treatment. Furthermore, if it does not spontaneously erupt, it represents a major burden to be solved during orthodontic treatment [36].

The removal of the gingival operculum and therefore the surgical exposure of the second molar with eruptive delay represents an attempt to ease the eruption of the tooth. This intervention does not have the purpose of direct disinclusion of the tooth but placing the tooth in the most favorable conditions to physiologically reach the occlusal plane.

Data analysis suggests that in most cases, retained permanent second molars have the potential to spontaneously erupt after operculectomy. Based on these premises, operculectomy should always be considered prior to other more invasive therapeutic options (i.e., extraction and so forth). It is clear how important an early diagnosis of the eruptive problem is. This is because once the roots of the second non-erupted molar have completed their development, the chances of a conservative intervention decrease considerably [13,37].

In the case of primary retention, the relationships between the coronal follicle and the overlying tissue seem to play an important role among factors limiting tooth eruption: a follicle malfunction or the presence of gingival tissue anomalies within the coating (such as the presence of a fibrous formation over the retained tooth) can hinder eruption [3]. In the present study, removing the oral mucosa alone over retained molars was able to successfully solve the clinical issue in 93.3% (70/75) of cases against 10% (7/70) of the control group. Eruption of second molars was classified as successful if it obtained a good vertical position with the occlusal surface <2 mm from the occlusal plane. This value is in accordance with the results obtained by Kenrad et al. (90% success rate) [38] and Nielsen et al. [39]. Second upper molars presented a higher success rate compared with the lower arch in both groups.

The advantage of operculectomy lies in its conservative nature: surgical exposure is a minimally invasive procedure and it is also useful in case operculectomy alone fails to obtain the treatment objective and orthodontic traction is required [40]. Patients were encouraged to maintain proper oral hygiene to allow the correct restoration of the periodontal support and underwent periodic follow-up visits. In treated cases, there were no complications following the operation and no periodontal problems occurred on treated teeth nor on first molars.

Results showed that the eruption of treated molars is more effective if they are vertically positioned or slightly mesially inclined. If the tooth has an altered axis of eruption, operculectomy itself shows poorer results, suggesting more complex causes need to be addressed to successfully treat patients. In such cases, early diagnosis is crucial and it allows to perform an adequate evaluation of posterior teeth crowding and to choose the best treatment option. Cone beam computed tomography (CBCT) allows a three-dimensional evaluation of the malocclusion making it easier to correctly diagnose and treat these kind of issues [41,42,43]. The decision to extract the molar and to plan an implant-supported rehabilitation should only be considered when operculectomy and other less invasive treatment options fail [44].

A recent retrospective study [45] evaluated several possible treatments of retained second molars. Even if all second molars treated with operculectomy reached the treatment objectives, no statistically significant conclusion was drawn due to the limited number of cases treated with it (10). The results of the aforementioned study are in agreement with the ones found by our study that, however, differ in the larger number of teeth treated with operculectomy, thus being able to reach a statistically significant difference between periodic non-interventional follow-up visits and surgical exposure. The limit of our study is the analysis of only two treatment methods. However, most of the already published studies evaluated four or more different treatments but failed to obtain statistical significance because of a smaller sample size per each treatment option [38,45]. Further studies are needed in the future on more treatment groups that analyze a higher sample size.

## 5. Conclusions

There are currently no reliable guidelines for the treatment of eruptive disturbances of retained second molars. Surgical exposure is a simple, minimally invasive and reliable method which allows in most cases the eruption of second molars. This treatment option does not preclude further more complex treatments and aims to ease spontaneous eruption. There is a statistically significant difference in the success rate of patients treated with operculectomy compared with controls.

## Figures and Tables

**Table 1:** Percentages of eruption of the second permanent molars, in the treatment group and in the control group.

Outcome	Treatment Group	Control Group
Second molars that have erupted (mean between upper and lower arch)	93.3% (*n* = 70)	10.2% (*n* = 6)
Upper second molars that have erupted	95.2% (*n* = 40)	8.5% (*n* = 3)
Lower second molars that have erupted	90.9% (*n* = 30)	8.5% (*n* = 3)
Odds ratio between case and control groups	10	1
*p*-value ^1^	*p* < 0.001	

^1^ Chi-squared test was used to compare the results of treatment group and untreated control group. Statistical significance was set at *p* value < 0.05.
